# Structural Basis for Group B *Streptococcus* Pilus 1 Sortases C Regulation and Specificity

**DOI:** 10.1371/journal.pone.0049048

**Published:** 2012-11-08

**Authors:** Roberta Cozzi, Daniil Prigozhin, Roberto Rosini, Francesca Abate, Matthew J. Bottomley, Guido Grandi, John L. Telford, C. Daniela Rinaudo, Domenico Maione, Tom Alber

**Affiliations:** 1 Novartis Vaccines and Diagnostics, Siena, Italy; 2 University of California, Berkeley, United States of America; University of Kansas Medical Center, United States of America

## Abstract

Gram-positive bacteria assemble pili through class C sortase enzymes specialized in polymerizing pilin subunits into covalently linked, high-molecular-weight, elongated structures. Here we report the crystal structures of two class C sortases (SrtC1 and SrtC2) from Group B *Streptococcus* (GBS) Pilus Island 1. The structures show that both sortases are comprised of two domains: an 8-stranded β-barrel catalytic core conserved among all sortase family members and a flexible N-terminal region made of two α-helices followed by a loop, known as the lid, which acts as a pseudo-substrate. *In vitro* experiments performed with recombinant SrtC enzymes lacking the N-terminal portion demonstrate that this region of the enzyme is dispensable for catalysis but may have key roles in substrate specificity and regulation. Moreover, *in vitro* FRET-based assays show that the LPXTG motif common to many sortase substrates is not the sole determinant of sortase C specificity during pilin protein recognition.

## Introduction

In recent years covalently-linked pilus-like structures have been identified as significant virulence factors on the surface of many Gram-positive bacteria including GBS [1]–[5]. Pilus structures mediate attachment to human epithelial cells [4], [6], contribute to GBS adherence to brain endothelium [7] and promote trans-epithelial migration [3]. Moreover, the pili extending from the surface of GBS have also been characterized as promising vaccine candidates [8], [9].

The pilin subunits of GBS are assembled into high molecular weight polymers via a transpeptidation reaction catalyzed by specific pilin-associated class C sortases, through a common mechanism involving specific motifs present in the pilin proteins [5], [10]–[14]. A C-terminal LPXTG-like motif (where X represents any amino acid), typically conserved in cell wall-anchored proteins, is present in the pilus structural subunits and represents the main sortase recognition site [4], [15], [16]. The pilin-related sortases, which are integral membrane cysteine transpeptidases, cleave the LPXTG-like motif of the pilin proteins and, via the Thr residue, covalently join the C-terminus of one pilin subunit to a Lys side chain (ε amino group) on the next subunit [15], [16]. In GBS and *C. diphtheriae*, the cell-wall anchoring of polymerized pili is mediated by the housekeeping class A sortase and the minor ancillary pilin, acting as the terminal subunit [11], [17], [18].

**Figure 1:**
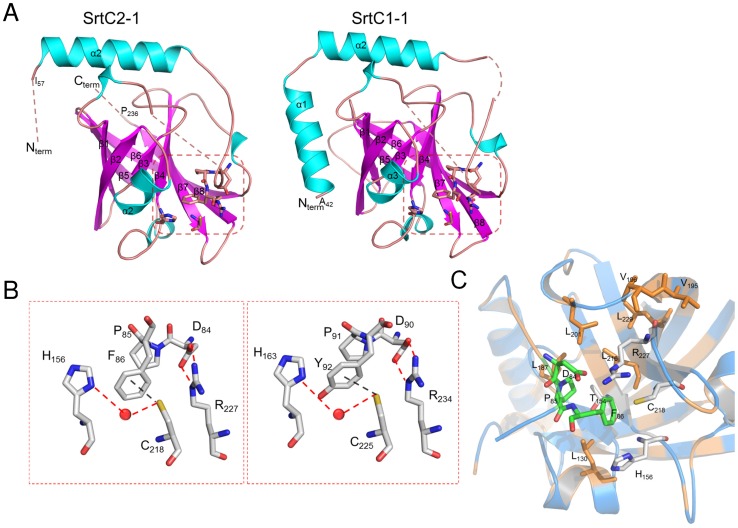
Overall fold of GBS PI-1 SrtC1 and SrtC2 and active site organization. (A) Overall fold of SrtC2 and SrtC1. Residues linking the mobile lid to the second helix and to the first beta-strand are missing in the final structures because of poor electron density, and are shown here as dashed lines. (B) Active sites of SrtC2 and SrtC1. Residues forming the mobile lid (Asp84-Phe86 in SrtC2 and Asp90-Tyr92 in SrtC1) and the active site (H156, C218, R227 in SrtC2 and H163, C225, R234 in SrtC1) are shown as sticks where sulfur, oxygen, and nitrogen atoms, are depicted as yellow, red, and blue, respectively. Water molecules are shown as red spheres. (C) The DPX motif is proximal to the catalytic triad of SrtC2, which is surrounded by conserved hydrophobic residues shown as sticks, where carbon, oxygen, and nitrogen atoms, are depicted as salmon, red, and blue, respectively.

Comparative analyses of the genome sequences of eight GBS strains has led to the identification of three genomic pilus islands named pilus island 1 (PI-1), 2a (PI-2a) and 2b (PI-2b), each with a similar genetic organization. Each pilus island codes for a main structural protein, known as the backbone protein (BP), two ancillary proteins (APs), and at least two pilus-associated sortase enzymes (SrtC1 and SrtC2) that are required for pilus protein polymerization [4], [19]. Genetic studies of the PI-1 and PI-2a loci established the relative contribution of sortases SrtC1 and SrtC2 in pilus assembly [19]. SrtC1 and SrtC2 enzymes were found to have some preference in terms of which ancillary protein (AP) they incorporate, as SrtC1 was more active in incorporating AP2, and SrtC2 preferentially incorporated AP1, while they both efficiently polymerized the backbone protein *in vivo* [4], [19]. PI-1 carries an additional gene predicted to code for a third sortase C enzyme (SrtC3) which is not essential for pilus assembly [20]. The co-expression of multiple pili has been observed [19], which can confer advantages given by each specific pilus, for example, only pilus type 2a was shown to have a specific role in GBS biofilm formation (PI-2a) [2]. Since pili components play such important roles in GBS pathogenesis, we focused our studies on providing further mechanistic insight into the molecular and structural basis of sortase-pilus protein recognition.

The crystal structures of several pilin-related class C sortases, including three sortases from *S. pneumoniae* [21], [22], AcSrtC-1 from *Actinomyces oris* [23], SrtC1 from *S. suis* [24] and GBS [25]–[27], have been reported. These structures all reveal a core 8-stranded beta-barrel, with the catalytic triad (His, Cys, Arg) situated in the active-site at the end of a groove along one side of the β barrel. The GBS and S. *suis* SrtC1 structures were determined with the active-site in the ‘open’ conformation, while the other structures showed the active site occluded by a loop region, termed the lid. The lid in SrtC1 from GBS PI-2a (SrtC1-2a) and *Actinomyces oris* SrtC2 is dispensable for sortase activity *in vivo* [27], [28]. Even better, kinetic characterizations of the cleavage activity of SrtC1-2a lid mutants on a peptide mimicking its substrate, the backbone protein of PI-2a (BP-2a), indicate that the mutants lacking the lid have higher activity than the wild type protein [27]. Based on the structures of GBS PI-1 SrtC1 crystallized in three different space groups, it was hypothesized that lid displacement is essential to promote substrate accessibility to the active site, while the catalytic Cys is available for interactions even when the lid covers the active site [25].

**Table 1:** Data Collection and Refinement Statistics.

	Sortase C1 PI-1	Sortase C2 PI-1
**Data Collection**		
Wavelength (Å)	1.12	1.12
Temperature (K)	100	100
Space group	P 1	P 4_1_ 2_1_ 2
Cell parameters		
*a b c* (Å)	38.98 48.47 59.37	60.44 60.44 102.24
α β γ (^o^)	87.31 76.85 72.64	90 90 90
Copies per a.s.u.	2	1
Resolution (Å) a	46.25−1.87 (1.93−1.87)	30.22−1.80 (1.86−1.80)
R_sym_ (%)	6.0 (30.8)	7.6 (57.6)
I/σI	25.7 (4.69)	17.8 (2.2)
Completeness (%)	80 (51)	97 (87)
Redundancy	6.0 (5.6)	6.6 (4.7)
**Refinement**		
Resolution (Å)	46.25−1.87^b^	30.22−1.80
Number of reflections	31,194	17,187
R_work_/R_free_ (%)	17.2/20.2	21.2/25.1
Number of atoms		
Protein	3161	1370
Solvent	350	91
B factors		
Protein (Å^2^)	28	32
Solvent (Å^2^)	34	34
Rmsd from ideal values		
Bond lengths (Å)	0.007	0.007
Bond angles (°)	1.025	1.088
Ramachandran plot		
Favored (%)	98	98
Allowed (%)	100	100
PDB ID	4G1J	4G1H

a Values in parentheses are for the highest resolution shell.

b Less than 50% of reflections were collected in 1.87−1.75 Å shell and used in refinement.

In this work, we present the high-resolution crystal structures of SrtC2 and SrtC1 from GBS PI-1. Structural analyses of these two sortases, coupled with biochemical assays, provide insights into the regulation and specificity of this family of enzymes.

## Results

### GBS PI-1 SrtC1 and SrtC2 Exhibit the Typical Sortase C Fold

The crystal structures of GBS SrtC1 and SrtC2 from PI-1 were determined at 1.87-Å and 1.8-Å resolution, respectively (Table 1, Figure 1A). Both structures were solved by molecular replacement using a poly-alanine model of *S. pneumoniae* SrtC1 (PDB 2W1J) [22] as a search template, with which GBS SrtC1 and SrtC2 both share 55% sequence identity. The refined model of GBS SrtC1 includes residues 42–263 for chain A and 43–246 for chain B. The refined model of SrtC2 includes residues 56–236; the first N-terminal residues 41–55, and the C-terminal residues 237–256, were not visible in the electron density maps. Although SrtC1 crystallized as a dimer in the asymmetric unit, the dimer interface is only 615 Å^2^, suggesting it is not physiologically relevant. Analytical size-exclusion chromatography under near physiological conditions (pH 7.5, 75 mM NaCl) indicated that both enzymes are monomeric in solution (Figure S1A), even at high (0.5–1 mM) concentration.

**Figure 2:**
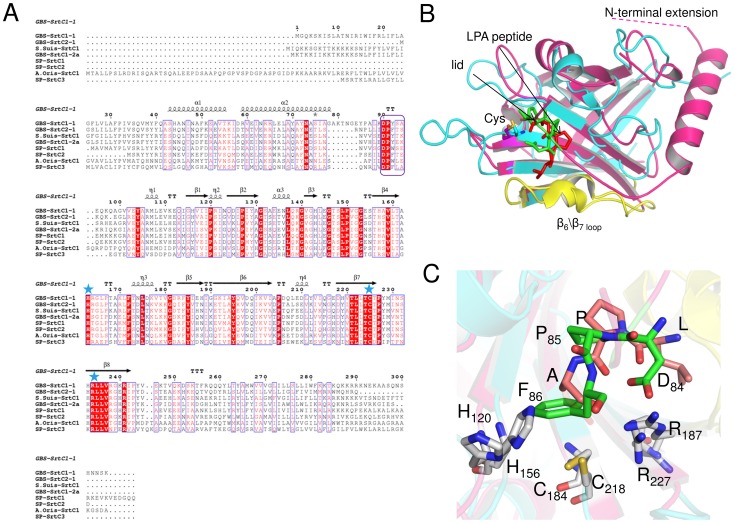
Structural comparison of GBS PI-1 SrtC2 crystal structure with *S. aureus* SrtA NMR structure. (A) Multiple sequence alignment of the pilus-forming sortases crystallized so far. Structure-based sequence alignment of the GBS PI-1 SrtC2, SrtC1, GBS PI-2a SrtC1 (PDB 3O0P), *S. pneumoniae* sortase C1 (PDB 2W1J), sortase C2 (PDB 3G69), sortase C3 (PDB 2W1K), *S. suis* SrtC1 (PDB 3RE9) and *A. oris* SrtC1 (PDB 2XWG). Identical residues are shown with a red background, whereas similar residues are shown in red and highlighted with blue boxes. Residues located within the active site cleft are conserved among all sortases and are highlighted with blue stars, whereas the lid residues DPF\Y\W are highlighted with a purple box (http://espript.ibcp.fr/ESPript/ESPript/). (B) Superposition of GBS PI-1 SrtC2 (pink) with the *S. aureus* SrtA (cyan, PDB 2KID) in complex with the sorting signal analogue LPAT* (red). The β6/β7 loops are colored in yellow. Unique structural features of SrtC enzymes are the N-terminal extension and the flexible lid (with the DPF motif in green as stick representation). The catalytic cysteine residues are highlighted in yellow and shown in a stick representation. The apo-SrtC2 structure overlaps with the structure of peptide-bound SrtA with an overall Ca rmsd of 2.33 Å for 118 aligned residues. (C) The lid residues DPF (color, Asp84, Pro85, Phe86) of GBS pilus-forming sortase is located analogously to the LPA substrate peptide (shown in sticks where carbon, oxygen, and nitrogen atoms, are depicted as white, red, and blue, respectively) in the structure of SrtA.

The overall fold of GBS PI-1 SrtC2 is similar to SrtC1, with a root-mean-square deviation (rmsd) of 0.94 Å for 169 aligned Cα atoms. SrtC2 also is similar to other pilus-associated sortases of *S. pneumoniae*, with rmsds of 1.2–2.2 Å (PDB entries 2W1J, 3G69 and 2W1K). Other homologues of GBS SrtC2 include: GBS SrtC1 of PI-2a (PDB 3O0P, rmsd 1.3 Å) and GBS Sortase A (PDB 3RBI, rmsd 1.5 Å). Both SrtC1 and SrtC2 exhibit an eight-stranded β-barrel structure, with the β-sheet core being surrounded by 2 major α helices in SrtC1 but only one α-helix in SrtC2, followed by a flexible loop, the lid, that sterically blocks the active site (Figure 1A–B). Like SrtC1, SrtC2 is predicted to form two N-terminal α-helices (Figure 2A); absence of electron density for the first α-helix of SrtC2 is likely an artifact of crystallization that may be due constraints imposed by crystal packing or simply to local disorder in this N-terminal region of the structure, as commonly observed in crystal structures. The His156, Cys218, Arg227 catalytic triad in SrtC2 is conserved in spatially equivalent positions in all sortase enzymes (Figure 2A). In GBS SrtC2 and SrtC1, the conserved catalytic His in the β4-α4 loop interacts indirectly with the catalytic Cys through the formation of a water-mediated hydrogen bond (Figure 1B).

The lid is present only in pilus-related sortases and contains a conserved DPX motif, where X can be any aromatic residue (Figure 2A). The side-chain carboxylate group of Asp84 in the lid of SrtC2 makes a salt bridge with the side chain of the conserved catalytic Arg227 (Figure 1B), and the ring of Phe86 interacts with Thr154 in a hydrophobic pocket made of conserved residues (Figure 1C and 2A). The pyrollidine ring of the conserved Pro85 in the lid also interacts with this hydrophobic pocket. GBS SrtC1 shows an identical arrangement of the catalytic pocket except for the Thr rotamer and the replacement of Phe86 with Tyr92 (Figure 1B). The aromatic ring of Phe86 in SrtC2 forms an aromatic-sulfur interaction with the catalytic Cys that has been previously observed in other pilus-related sortases (Figure 1B) [21], [22], [27]. The lid residues 39–44 and 53–60 of SrtC1, and 89–95 of SrtC2, could not be modeled due to a lack of electron density, while the conserved residues of the DPX motif were well ordered in both structures.

**Figure 3:**
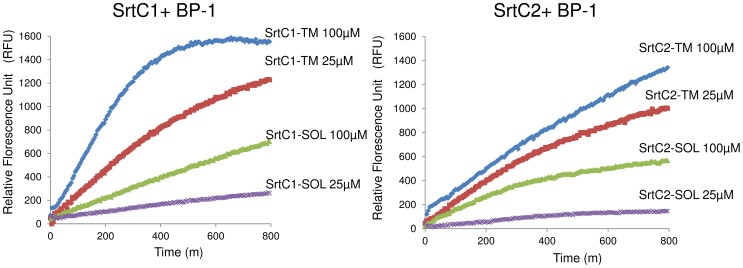
Enzymatic activity of SrtC1 and SrtC2 with or without the C-terminal transmembrane (TM) region. FRET assay of sortase enzymes SrtC1_42–263_ (SrtC1-SOL) and SrtC2_42–256_ (SrtC2-SOL), lacking the leader sequence, the N-terminal and C-terminal hydrophobic regions, in comparison with SrtC1_42–305_ (SrtC1-TM) and SrtC2_42–283_ (SrtC2-TM), preserving the C-terminal TM region and lacking the leader sequence and the predicted N-terminal hydrophobic region using the fluorescent peptide BP-1 (128 µM) containing an LPXTG motif as a substrate. Each recombinant enzyme was analyzed at 25 µM and 100 µM and the fluorescence emission was monitored every 5 minutes. The higher values in fluorescence intensity were observed with SrtC1-TM and SrtC2-TM.

### The Sortase C Lid is a Pseudo-substrate

To investigate substrate binding in sortase C enzymes, the crystal structures of SrtC1 and SrtC2 were superimposed on the NMR structures of apo and substrate-bound *S. aureus* sortase A (SrtA) (PDB 1IJA and 2KID) [29], [30]. This analysis showed that GBS sortase C enzymes exhibit the same fold as *S. aureus* sortase A. The β barrel structural core is conserved in the GBS SrtC and *S. aureus* SrtA enzymes (Figure 2B); for example, Cα rmsd of 1.9 Å aligning SrtA with GBS SrtC2. Unlike SrtA, GBS sortase C enzymes contain an additional N-terminal extension, composed of one (SrtC2) or two (SrtC1) α helices and a lid that blocks access of substrates to the active site (Figure 2B, 2C and S2). Surprisingly, the ligand-free SrtC structures matched the active, peptide-bound, conformation of *S. aureus* SrtA better than that of apo-SrtA. For both GBS SrtC1 and SrtC2 structures, the lid overlapped the LPAT peptide that is covalently bound to the catalytic Cys in the catalytic pocket of *S. aureus* SrtA. In particular, the conserved motif DPY or DPF in the SrtC lids overlap with the LPAT peptide, with the proline in a similar position in both the lid and the peptide. These observations suggest that the conserved residues in the lid that interact with the active site of GBS sortases are pseudo-substrates, insofar as they mimic the binding of the LPXTG motif in the catalytic site.

**Figure 4:**
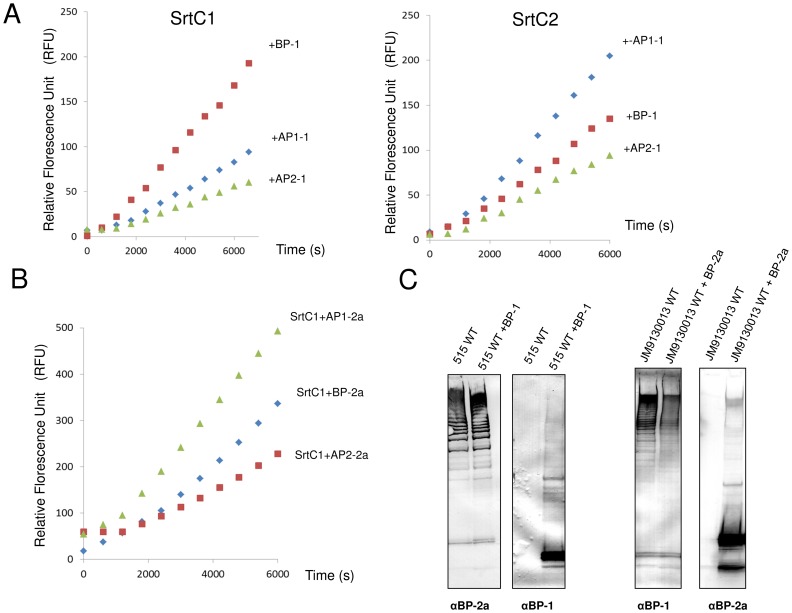
FRET assay with PI-1 and PI-2a peptides for substrate specificity analysis of PI-1 SrtC1 and SrtC2. (A) The reaction solutions contained 128 µM of PI-1 fluorescent peptides and 25 µM of SrtC1-TM (left panel) or SrtC2-TM (right panel). The reactions were performed at 37°C in the assay buffer containing 25 mM HEPES pH 7.5, 100 mM NaCl and 1 mM DTT. Fluorescence emission was monitored every 10 minutes and an increase in fluorescence intensity was observed in the presence of BP, AP1 and AP2 peptides mimicking the LPXTG motif of PI-1 pilus proteins. (B) Reactions were performed with PI-2a peptides and 25 µM of SrtC1-TM in the same conditions described above. (C) *In vivo* substrate specificity analysis. Immunoblots of total protein extracts from GBS 515 (containing SrtC1 and SrtC2 of pilus island 2a) and JM9130013 (containing SrtC1 and SrtC2 of pilus islands 1 and 2b) wild-type and complemented strains with plasmids expressing the backbone proteins of PI-1 (BP-1) and PI-2a (BP-2a), respectively. The nitrocellulose membranes were probed with antisera specific for BP-1 and BP-2a.

### Recombinant PI-1 SrtC1 and SrtC2 are Catalytically Active

To measure the catalytic activity of recombinant GBS PI-1 SrtC1 and SrtC2, we utilized a FRET (Foerster Resonance Energy Transfer)-based assay to monitor cleavage of a self-quenched fluorescent peptide (which only emits significant fluorescence upon cleavage) containing the LPXTG-like motif of the backbone protein of PI-1 (BP-1) [17], [27]. For several sortases including SrtC1 from PI-2a, mutants lacking one or both transmembrane (TM) domains have lower enzymatic activity than the wild-type enzyme [27], [31], [32]. Similarly for SrtC1 and SrtC2, we found that the soluble domains (SrtC1-SOL and SrtC2-SOL) were less active than constructs containing the entire C-terminal TM region (SrtC1-TM and SrtC2-TM) (Figure 3). Consequently, we used SrtC1-TM and SrtC2-TM for the following *in vitro* experiments.

**Table 2:** Sequences of fluorescent peptides used in this study.

Name	Sequence
BP-1	Dabcyl-RPS**IPNTG**GIG-Edans
AP1-1	Dabcyl-RPPGV**FPKTG**GIG-Edans
AP2-1	Dabcyl-RGGL**IPKTG**EQQ-Edans
BP-2a	Dabcyl-KKVT**IPQTG**GIGT-Edans
AP1-2a	Dabcyl-KGI**IPKTG**GK-Edans
AP2-2a	Dabcyl-SF**LPKTG**M-Edans

### The LPXTG-like Motif is not the only Determinant of SrtC Specificity

Although genetic studies of GBS PI-1 SrtC1 and SrtC2 showed a certain level of cross-specificity, SrtC1 was most effective in polymerizing ancillary protein 2 (AP2), while SrtC2 preferred AP1 [19]. The enzymatic activity of GBS PI-1 SrtC1 and SrtC2 was tested on fluorescent peptides mimicking the LPXTG-like motifs of BP, AP1 and AP2 from PI-1 (Table 2) in FRET assays. We observed that both sortases can cleave the three LPXTG-like peptides tested although the BP and AP1 peptides were more efficiently hydrolyzed compared to AP2 peptide (Figure 4A). To further characterize specificity and substrate recognition in GBS PI-1 SrtC1 and SrtC2, we measured steady state kinetic parameters for PI-1 peptides hydrolysis by the two sortase enzymes at different substrate concentrations (Figure S3) and we compared the V_max_, Km and Kcat\Km parameters (Table 3). The kinetic parameters of SrtC1 and SrtC2 were similar; both enzymes resulted more efficient in processing AP1 peptide with the lower Km values (3.58 µM for SrtC1 and 6.38 µM for SrtC2) compared to the Km values obtained for BP (31.00 µM for SrtC1 and 21.56 µM for SrtC2) and AP2 peptides (16.39 µM for SrtC1 and 27.33 µM for SrtC2). Moreover the highest Kcat/Km values (3.25×10^−4^ µM^−1^s^−1^ for SrtC1 and 1.62×10^−4^ µM^−1^s^−1^for SrtC2) were obtained with the AP1 peptide. However, SrtC1 showed the highest V_max_ value for BP mimicking peptide (4.44×10^−2^ µM s^−1^) while SrtC2 for the AP1 peptide (2.60×10^−2^ µM s^−1^).

Moreover, we tested the hydrolyzing activity of SrtC1 and SrtC2 on peptides containing the LPXTG-like motifs of the pilus structural subunits belonging to GBS PI-2a (Table 2). Both PI-1 SrtC1 and SrtC2 hydrolyzed all three PI-2a peptides analyzed (Figure 4B). Since both PI-1 C sortases recognize the LPXTG-like motif of the pilin substrate of both PI-1 and PI-2a *in vitro*, we hypothesized that the sortases of one pilus could polymerize pilus proteins of other pili.

To investigate the biological significance of our findings, we performed *in vivo* complementation studies of GBS strain JM9130013, expressing both PI-1 and PI-2b, but lacking SrtC of PI-2a and strain 515, expressing only PI-2a components. As revealed by Western blot analysis, the over-expression of PI-2a backbone protein (BP-2a) in JM9130013 strain and PI-1 backbone protein (BP-1) in 515 strain resulted in the formation of hybrid high molecular weight structures (Figure 4C). However, the incorporation of both proteins into pili, mediated by heterologous C sortases belonging to a different pilus island, resulted less efficient than in the wild-type strains. Moreover, the majority of the over-expressed proteins remained in monomeric form (Figure 4C). These data, showing a partial substrate promiscuity of C sortases from different pilus islands, are in agreement with the activity shown *in vitro* by SrtC1 and SrtC2 from PI-1 on peptides containing the LPXTG sorting signal of both PI-1 (Figure 4A) and PI-2a (Figure 4B), suggesting that GBS sortase C enzymes can recognize several and different sorting signals.

**Table 3:** Kinetic constants Km, Vmax and Kcat of wild-type and mutant sortases.

**Enzyme**	**PI-1 Peptide**	**Vmax (µM s** ^−**1**^ **)**	**Km (µM)**	**Km** **Std. Error**	**Kcat (s** ^−**1**^ **)**	**Kcat** **Std. Error**	**Kcat/Km (µM** ^−**1**^ ** s** ^−**1**^ **)**
**SrtC1_WT_**	BP	4.44×10^−2^	31.00	±4.62	1.77×10^−3^	±1.01×10^−4^	5.70×10^−5^
**SrtC1_Y92A_**	BP	7.45×10^−2^	32.35	±6.07	2.98×10^−3^	±2.18×10^−4^	9.22×10^−5^
**SrtC1_ΔNT_**	BP	1.80×10^−1^	49.69	±6.43	4.32×10^−3^	±2.48×10^−4^	8.69×10^−5^
**SrtC1_WT_**	AP1	2.91×10^−2^	3.58	±0.63	1.16×10^−3^	±4.40×10^−5^	3.25×10^−4^
**SrtC1_Y92A_**	AP1	5.41×10^−2^	5.69	±1.00	2.16×10^−3^	±9.40×10^−5^	3.81×10^−4^
**SrtC1_ΔNT_**	AP1	7.45×10^−2^	6.98	±0.86	2.98×10^−3^	±9.40×10^−5^	4.27×10^−4^
**SrtC1_WT_**	AP2	1.92×10^−2^	16.39	±2.50	0.77×10^−3^	±3.80×10^−5^	4.70×10^−5^
**SrtC1_Y92A_**	AP2	2.83×10^−2^	14.21	±2.48	1.13×10^−3^	±6.10×10^−5^	7.97×10^−5^
**SrtC1_ΔNT_**	AP2	5.74×10^−2^	27.6	±3.54	2.30×10^−3^	±1.09×10^−4^	8.32×10^−5^
**SrtC2_WT_**	BP	1.83×10^−2^	21.56	±2.88	7.31×10^−4^	±3.33×10^−5^	3.39×10^−5^
**SrtC2_F86A_**	BP	2.66×10^−2^	43.85	±12.70	1.06×10^−3^	±1.31×10^−4^	2.43×10^−5^
**SrtC2_ΔNT_**	BP	1.39×10^−1^	57.15	±3.54	5.56×10^−3^	±1.74×10^−4^	9.72×10^−5^
**SrtC2_WT_**	AP1	2.60×10^−2^	6.385	±1.42	1.04×10^−3^	±5.82×10^−5^	1.62×10^−4^
**SrtC2_F86A_**	AP1	1.22×10^−1^	60.36	±7.95	4.90×10^−3^	±3.04×10^−4^	8.10×10^−5^
**SrtC2_ΔNT_**	AP1	1.24×10^−1^	16.07	±3.16	4.96×10^−3^	±3.10×10^−4^	3.09×10^−4^
**SrtC2_WT_**	AP2	1.09×10^−2^	27.33	±4.35	4.36×10^−4^	±2.56×10^−5^	1.59×10^−5^
**SrtC2_F86A_**	AP2	1.03×10^−2^	18.14	±2.97	4.10×10^−4^	±2.21×10^−5^	2.26×10^−5^
**SrtC2_ΔNT_**	AP2	6.15×10^−2^	18.14	±2.97	2.46×10^−3^	±1.32×10^−4^	1.35×10^−4^

### The Conserved β-barrel Core is Sufficient for LPXTG Cleavage

**Figure 5:**
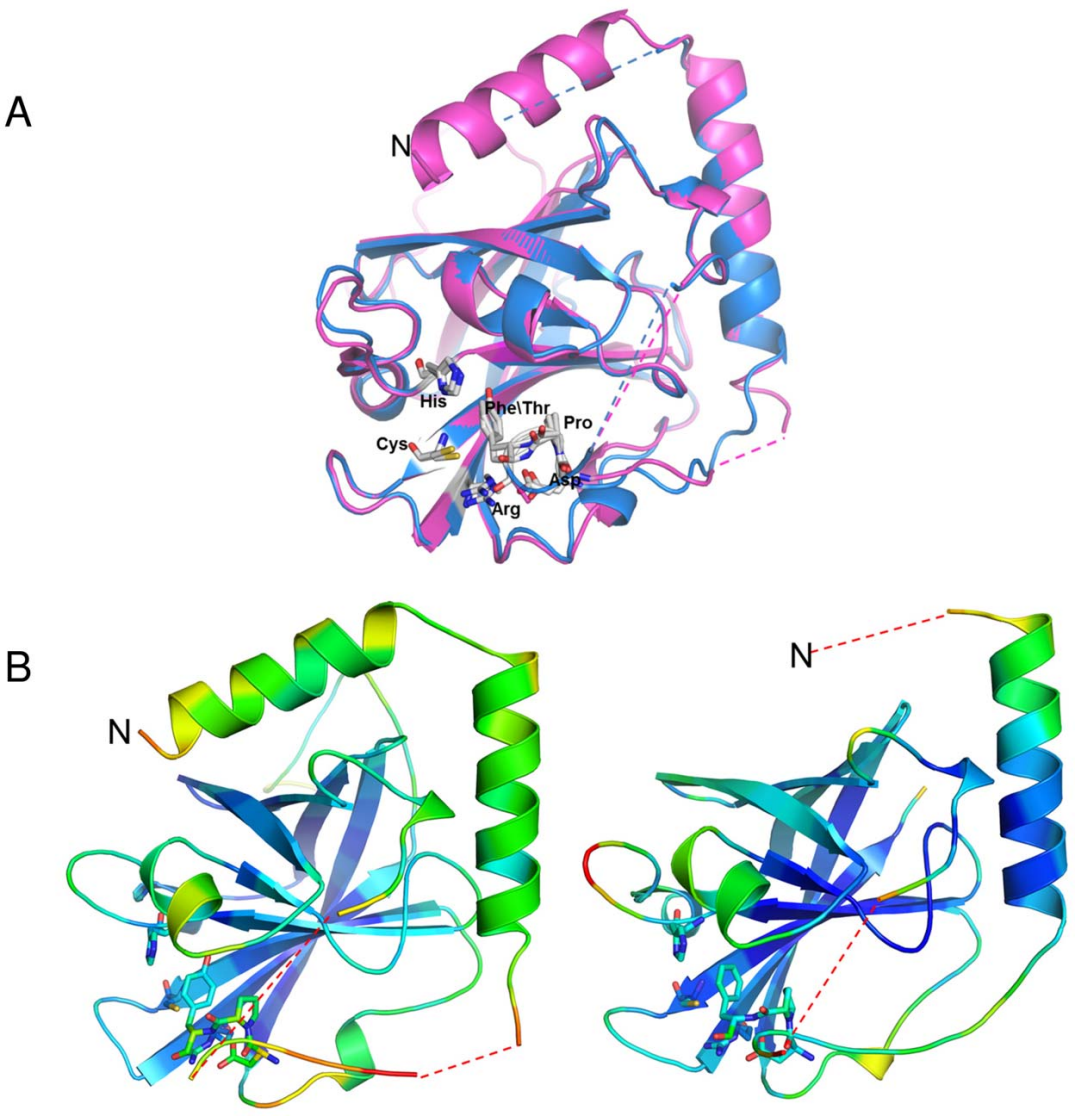
Structural analysis of GBS PI-1 SrtC enzymes. (A) Superposition of GBS PI-1 SrtC2 (in blue) with GBS SrtC1 (magenta). Residues forming the mobile lid (Asp90, Tyr92 in SrtC1 and Asp 84, Phe 86 in SrtC2) and the active site (H163, C225, R234 in SrtC1 and H156, C218, R227 in SrtC2) are shown as sticks. Localized lack of electron density in the loop regions of both structures suggests that the N-terminal region is flexible. (B) GBS PI-1 SrtC1 (left panel) and SrtC2 (right panel) ribbon diagrams colored according to the B-factor distribution, from low (blue) to high (red). The average residue B-factors range from 13.8 to 65.3 Å2 in SrtC1 and from 19.2 to 63.1 Å2 in SrtC2.

**Figure 6:**
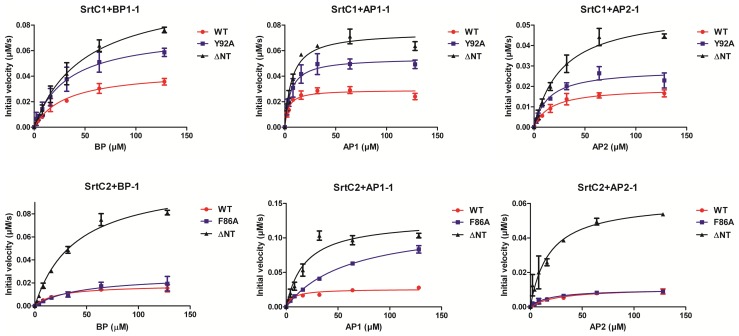
Kinetic analysis of PI-1 SrtC1 and SrtC2 wild-type and mutants. Triplicate data sets for each experiment were used to calculate the steady-state velocity at different PI-1 peptides concentrations for each enzyme and were expressed as initial rates (µM/s) versus concentration of substrate. SrtC1 (top) and SrtC2 (bottom) enzymes carrying the mutation Y92A and F86A (SrtC1_Y92A_ and SrtC2_F86A_) and the deletion of the entire N-terminal region (SrtC1_ΔNT_ and SrtC2_ΔNT_) were analyzed in comparison with wild-type enzymes by FRET assays at various concentrations of three different PI-1 peptides (Table 2). The reactions containing 25 µM of enzyme and 2–128 µM of fluorescent peptide were performed at 37°C in 20 mM HEPES pH 7.5, 100 mM NaCl and 1 mM DTT.

A lack of electron density suggested that the N-terminal region, including the interhelical loop, is flexible in both SrtC1 and SrtC2 (Figure 1A and Figure 5A). Additionally, the B-factors of residues in the N-terminal extension suggest (including residues 42–103 of SrtC1 and 56–96 of SrtC2, respectively) that this portion of sortase C enzymes is more mobile than the β-barrel core that is common to all sortase family members (Figure 5B). Our hypothesis, based on structural analysis of GBS SrtC1 and SrtC2, is that the entire N-terminal extension (comprised of the two α helices and the lid), but not the lid alone, may contribute to enzyme regulation. To test this hypothesis, we characterized truncated versions of SrtC1 and SrtC2 in which the entire N-terminal regions (61 and 54 residues, respectively) were removed (SrtC1_ΔNT_ and SrtC2_ΔNT_). We tested their cleavage activity *in vitro* on fluorescent peptides mimicking the LPXTG-like motifs of BP, AP1 and AP2 from PI-1 in comparison to the wild-type enzymes and SrtC1_Y92A_ and SrtC2_F86A_ variants, which contain a substitution of the aromatic residue in the lid that interacts directly with the catalytic cysteine (Figure 1B). With all the three peptides tested, both SrtC1_ΔNT_ and SrtC2_ΔNT_ showed the highest activity compared to SrtC1_Y92A_ and SrtC2_F86A_ lid mutants and wild type enzymes (Figure 6). However, the BP-1 and AP1-1 peptides appeared hydrolyzed more efficiently than the AP2-1 peptide. The estimated V_max_ values for LPXTG-like peptides cleavage reactions confirm that SrtC1_ΔNT_ and SrtC2_ΔNT_ efficiently cleave all the peptides tested, with an increase of V_max_ values of even 10-fold respect to the wild-type SrtC1 and SrtC2 and SrtC1_Y92A_ and SrtC2_F86A_ lid mutants (Table 3). Thus, the entire N-terminal, containing the α-helices and the entire lid loop, is not required for the SrtC catalytic activity, rather its deletion clearly enhance the enzyme activity.

## Discussion

In Gram-positive bacteria, pilus biogenesis occurs through a two-step mechanism, where pilin subunits are polymerized into high molecular weight (HMW) structures by class C pilin-specific sortases and are then covalently anchored to the cell wall by the housekeeping sortase [17], [18], [33]. The two steps of the pilin polymerization reaction require different signals. First, sortase C enzymes recognize and cleave the LPXTG motif of the pilin protein, forming an acyl-enzyme intermediate. In the second step, the intermediate is resolved upon nucleophilic attack by Lys in the pilin motif of the next pilin subunit. Vengadesan *et al.* reviewed the model of GBS P-1 assembly with the incorporation of the minor ancillary protein (AP2) at the base of the pilus and the major ancillary (AP1) at the tip, in according to the general model proposed for S. *pneumoniae* and C. *diphtheriae* [12], [34], [35].

In this work, we determined the crystal structures of GBS PI-1 SrtC2 and SrtC1. In both enzymes, the catalytic residues are not accessible to pilin substrates, suggesting that the enzymes cannot bind substrates in this conformation. The super-imposition of the *S. aureus* SrtA structure with GBS Sortase C structures shows that the entire catalytic β-barrel structural core is conserved. Unlike SrtA, GBS sortase C enzymes contain an additional N-terminal extension of approximately 50 residues, composed of one or two α-helices and a lid that blocks the access of substrates to the active site. Surprisingly, ligand-free SrtC structures are more similar to the peptide-bound SrtA structure than to apo-SrtA. The structural similarity between the LPXTG peptide in the active site of SrtA suggests that the conserved residues in the lid that interact with the active site of GBS sortase act as a pseudo-substrate. This observation further supports the already proposed regulatory role played by the lid in restricting the access of the pilin substrates to the catalytic cleft [21], [22], [27].

The S. *aureus* SrtA does not contain an N-terminal extension or a lid and might represent the smallest sortase module retaining catalytic activity. Based on the high-resolution structures of GBS SrtC1 and SrtC2, truncated constructs were designed to produce the sortase core domain, devoid of the N-terminal region. Our structural analysis combined with *in vitro* experiments performed with fluorogenic peptides and with N-terminal deletion mutants of SrtC1 and SrtC2 show that the entire N-terminus, and not just the lid, as shown for GBS PI-2a SrtC1 [27], is disposable for catalysis. Thus, the minimum active sortase region is the β-sheet core seen in the *S. aureus* SrtA structure and common to all sortase family members. The N-terminal extension is a unique feature of class C sortases and appears to function as a regulatory motif. Both class A and class C sortases cleave LPXTG-like motifs, but only sortase C can polymerize the pilus proteins to form high molecular weight structures. Hence, the different function of SrtC compared to SrtA, in terms of regulation, specificity or localization, may be due to the presence in this specific class of enzymes of a highly specialized N-terminal segment. Moreover, compared to the conserved central β-barrel core harboring the active site, except for the DPY\W\F motif, this N-terminal extension that includes the helices and the lid is the most variable sequence region in Gram positive sortases (Figure 2A). Based on these analyses, we propose that the SrtC enzymes can be considered as having two functional domains: (i) an N-terminal regulatory region that contains the flexible inhibitory, pseudo-substrate lid, involved in enzyme regulation and probably specificity; and (ii) an enzymatic region, the β-barrel core that contains the catalytic triad.

A characteristic feature of class C sortases is the presence of a C-terminal transmembrane domain. Sortases lacking this domain are not able to polymerize pili *in vivo*, as it is essential for the enzyme insertion into the membrane, or to cleave synthetic mimicking peptides *in vitro* [27], [28]. So far, no crystal structure has been determined for full-length class C sortases. Therefore, alternative approaches will be necessary, to clarify the structure-function relationship of this transmembrane domain.

Our *in vitro* and *in vivo* complementation studies revealed that both GBS PI-1 C sortases can cleave all the LPXTG-like peptides tested exhibiting a functional promiscuity for pilin subunit incorporation into pili. However, the observed substrate promiscuity is not surprising, since it had been previously reported for other Gram positive sortase C enzymes, including the pilus-associated sortases in *C. diphtheriae* and *S. pneumoniae* [36]–[38]. Apparently conflicting results were obtained by genetic studies in GBS strains expressing pilus type 1 or 2a [19]. In these studies each class C enzyme, although clearly exhibited redundant functions, predominantly incorporated into pili one of the two ancillary subunit, with significantly reduced ability to incorporate the other pilin. Taken together, these studies suggest that the promiscuous action shown by class C sortases on distinct substrates originates from their ability to cleave variable LPXTG-like motifs. On the other hand, the preferential ancillary protein incorporation observed *in vivo,* not apparent in the cleavage reactions with peptides *in vitro*, suggests that the substrate specificity of C sortases may be due to recognition of more extensive structural determinants rather than a few specific residues. Interestingly, the lower enzyme activity displayed in *in vitro* assays (Figure 4 and Table 3) by both SrtC1 and SrtC2 on the AP2 peptide can be explained as the LPXTG-like motif of the minor ancillary protein has been demonstrated to be substrate of SrtA for anchoring the entire polymerized pilus to the cell wall [17], [18]. In this context, the specificity of SrtC1 for AP2 observed *in vivo* [19], but not detectable with our LPXTG-peptides based assay, can be due to the specific joint of the lysine residue in the AP2 pilin motif to the threonine residue within the LPXTG-like motif of the BP. Our data suggest that *in vitro* experiments involving only the sortases in combination with LPXTG-like mimicking peptides are likely to be insufficient to define the determinants of sortase C enzyme specificity. There are obviously other factors, *in vivo*, in addition to the LPXTG-like motif, that guide sortase C specific substrate recognition.

The crystal structures of the two PI-1 SrtC enzymes suggest that the hydrolysis of different LPXTG-like peptides may be a consequence of the conservation of the residues and the β-sheet fold of the catalytic domain and of the flexibility of the entire N-terminal domain that could allow LPXTG-like peptides to bind productively to the catalytic cleft.

Further experiments involving the sortase, LPXTG motif and pilin motif-containing proteins will be required in order to understand fully the molecular basis of substrate specificity, which in turn may determine variations in pilus assembly, virulence and pathogenesis.

## Materials and Methods

### Ethics Statement

Animal treatments were performed in compliance with the Italian laws, and approved by the institutional review board (Animal Ethical Committee) of Novartis Vaccines and Diagnostics, Siena, Italy.

### Bioinformatics

Transmembrane helices and membrane topology of sortase protein sequences were predicted using *TMHMM* [39]. Multiple sequence alignments were performed using *ClustalW* [40].

### Cloning, Expression and Purification of Recombinant Proteins

Gene fragments coding for GBS SrtC1 and SrtC2 (TIGR annotation SAG_0647 and SAG_0648) were PCR amplified from GBS strain 2603V/R. PCR fragments encoding GBS SrtC1_42–263_ and SrtC2_41–256_ were cloned using ligation independent cloning into the 2BT vector (MacroLab) to generate N-terminally His-tagged proteins. Proteins were expressed in *E. coli* Rosetta™(DE3) pLysS cells (Novagen). The cells were grown at 37°C in Luria Broth to an optical density OD600 of 0.7 and induced with 0.5 mM IPTG for 5 hr at 25°C. The soluble proteins were extracted by French press in 25 mM Hepes (pH 7.5), 400 mM NaCl, 20 mM imidazole, 10% Glycerol, 5 mM beta-mercaptoethanol (BME), lysozyme and protease inhibitors and purified by a FF-Crude His-Trap HP nickel chelating column (Amersham Bioscience) followed by a desalting column in 25 mM HEPES (pH 7.5), 400 mM NaCl, 20 mM imidazole, 10% glycerol, 5 mM beta-mercaptoethanol (BME). The His-tag was cleaved with AcTEV protease, and then removed by a subtractive IMAC purification step. The monomeric state of recombinant SrtC1 and SrtC2 was assessed by gel filtration using an S75 10 300 column in 25 mM HEPES (pH 7.5), 75 mM NaCl, 0.5 mM TCEP, 5% glycerol. Purity was checked by SDS-PAGE. A Mosquito crystallization robot was used for screening commercially available sets of crystallization reagents.

For FRET assays, the SrtC1 and SrtC2 enzymes were cloned and expressed as His-MBP (Maltose Binding Protein) fusion proteins in order to promote solubility. The mutants SrtC1_Y92A_, SrtC2_F86A_ and SrtC1_ΔNT_ and SrtC2_ΔNT_ (including residues 103–305 of SrtC1 and 96–283 of SrtC2, respectively) were generated by PIPE mutagenesis [41] using as template the His-MBP-SrtC1_42–305_ and His-MBP- SrtC2_42–283_ constructs. ΔLID enzymes, SrtC1_Δ86–102_ and SrtC2_Δ79–95_, with the deletion of the lid region, were also produced, but they expressed as insoluble enzymes and, consequently, they could not be included in our study. The enzymes fused with His-MBP were extracted after re-suspending 2 g of cell pellet in 25 mL of Cell Lytic express solution (Sigma) containing detergents, lysozyme and DNAse and purified by a FF-Crude His-Trap HP nickel chelating column and an MBP Trap HP column.

### Crystallization, Data Collection and Structure Determination

Crystals of SrtC1 (SAG_0647) were grown at 18°C by vapor diffusion in hanging drops containing equal volumes (1 µl) of 10 mg/ml of SrtC1 and a reservoir solution consisting of 0.4 M Sodium Formate, 0.1 M Bis-Tris propane pH 6.5, 22% PEG 3350. Crystals of SrtC1 belong to space group *P*1, and the asymmetric unit contains two protein molecules.

Crystals of SrtC2 (SAG_0648) were grown at 18°C by vapor diffusion from 0.26 M CaCl_2_, 19% PEG 6000, 0.1 M HEPES pH 7. Crystals of SrtC2 belong to space group *P41 21 2*, and the asymmetric unit contains one SrtC2 monomer.

Diffraction data were collected at 100 K on beamline 8.3.1 of ALS and processed by using *HKL2000* [42]. The structure of GBS SrtC1 and SrtC2 were solved by molecular replacement in *Phenix* [43] using as a search model poly-Ala coordinates of PDB entry 2W1J (55% sequence identity). The models were refined using *Phenix* [43] and *Coot* [44] and validated using *Molprobity* [45]. Chimera [46] and Pymol (http://pymol.org) were used for model analysis and illustrations.

### FRET Assay

To monitor the *in vitro* activity of recombinant SrtC1 and SrtC2, we used fluorescently self-quenched peptides (Thermo Scientific Biopolymers) tagged with Edans as fluorophore and Dabcyl as quencher, containing the LPXTG-motif BP, AP1 and AP2 subunits from PI-1 or PI-2a (Table 2). The hydrolysis of the peptides by sortases results in an enhanced fluorescence signal as the Edans group is separated from the quencher Dabcyl group. Activity tests were performed in triplicate in 25 mM HEPES buffer [pH 7.5], 100 mM NaCl, 1 mM DTT, 25 µM enzyme and 128 µM fluorogenic peptides. Reactions were started by the addition of enzymes and were monitored by measuring the increase in fluorescence every 10 minutes (λex = 336 nm, λem = 490 nm) at 37°C on an Infinite M200 Spectrophotometer microplate reader (TECAN). As controls, the peptides were incubated without the enzyme or with His-MBP alone and the RFU (Relative Fluorescence Units) values were normalized against the controls. The synthetic fluorogenic peptides were dissolved in 50% DMSO.

### Complementation of GBS Strains

Electro-competent GBS 515 and JM9130013 cells containing pilus island 2a (PI-2a) and pilus islands 1 and 2b (PI-1 and PI-2b) respectively, were transformed with the complementation vectors pAM-BP-2a and pAM-BP-1 described previously [19]. Complementation was confirmed by checking for protein expression by Western Blot.

### Antibodies and Western Blot

Antisera specific for the recombinant proteins were produced by immunizing CD1 mice with the purified proteins as described previously [9].

Western blot analysis was performed as described previously [27]. Briefly, mid-exponential phase bacterial cells were centrifuged and suspended in 50 mM Tris-HCl containing 40 U of mutanolysin (Sigma-Aldrich) and COMPLETE protease inhibitors (Roche). The mixtures were then incubated at 37°C for 1 h. Cellular debris were removed by centrifugation and protein concentration was determined using BCA protein assay (Pierce, Rockford, IL). Total protein extracts (20 µg) were resolved on 3–8% precast gels (Invitrogen) and transferred to nitrocellulose. Membranes were probed with mouse antiserum directed against structural pilus proteins (1∶1.000 dilution) followed by a rabbit anti-mouse horseradish peroxidase-conjugated secondary antibody (Dako, Glostrup, Denmark). Bands were then visualized using an Opti-4CN substrate kit (Bio-Rad).

### Kinetic Measurements

Kinetic experiments were performed by incubating various concentrations of peptides (ranging from 2 µM to 128 µM) with a constant enzyme concentration of 25 µM. As control, the same peptide concentrations were incubated without the enzyme. All reactions were performed at 37°C in 20 mM HEPES pH 7.5, 75 mM NaCl, 1 mM DTT and were initiated by the addition of enzyme and monitored by measuring the fluorescence increase every 10 minutes for 100 minutes (λex = 336 nm, λem = 490 nm) on an InfiniteM200 Spectrophotometer microplate reader (TECAN). Assays were carried out in 96 W black plates (Greiner).

Initial velocities (V) were determined from the progress curves and plotted against substrate concentration. Velocities were calculated as the difference of fluorescence values after 200 minutes versus the time (minutes), representing the initial rate of the reaction. The data were fitted to the Michaelis–Menten equation V  =  Vmax[S]/(Km+[S]) with a non-linear regression analysis program (GraphPad). The best fits of the data produced Vmax, Km and Kcat values that are reported in Table 3.

## Supporting Information

Figure S1 **SEC analysis of recombinant SrtC2 and SrtC1.** Recombinant enzymes run on a Superdex 75 10/300 SEC column connected to an Äkta Purifier. The Gel filtration standard (Biorad, 151-1901) was run on the same column and in the same buffer conditions: Thyroglobulin (670 kDa) elution volume 7.79 ml, γ-globulin (158 kDa) elution volume 8.10 ml, Ovalbumin (44 kDa) elution volume 9.92 ml, Myoglobin (17 kDa) elution volume 12.15 ml, Vitamin B12 (1.35 kDa) elution volume 18.17 ml. (A) The peaks at 11.77 ml and 10.97 ml correspond to monomeric SrtC2-SOL (predicted MW of 24 kDa) and SrtC1-SOL (predicted MW of 24.8 kDa) used for crystallization trials. (B) Superimposition of the chromatograms of soluble and TM containing sortases SrtC2-SOL/SrtC2-TM both fused with HIS-MBP (predicted MW 68.7 kDa and 71.4 kDa) and SrtC1-SOL/SrtC1-TM with HIS-MBP (predicted MW 69.5 kDa and 75.2 kDa), used for the FRET assays. The SrtC-TM proteins were prepared as HIS-MBP fusions in order to improve their solubility, as described in Materials and Methods. Here, the SrtC-SOL proteins were also prepared in HIS-MBP format, in order to allow a direct comparison with the SrtC-TM HIS-MBP proteins. Soluble sortases are mostly monomeric (blue chromatograms), eluting at the volumes of 9.33 ml and 9.19 ml. SrtC2-TM and SrtC2-TM (green chromatograms) eluting at the volumes of 7.92 ml and 7.88 ml are mostly aggregated, based on the standard.
(TIF)

Figure S2 **Superposition of the open-conformation (PDB 3RBJ) and close-conformation structures of GBS SrtC1-1 (in green) with** ***S. aureus*** **SrtA peptide-bound (red, PDB 2KID).** In the open-conformation structure of GBS PI-1 SrtC1, the lid is displaced from the active site and the cleft is free to accommodate the LPA peptide (red spheres). On the contrary, in the close-conformation structure the conserved motif DPY (green spheres) in the SrtC1 lid overlaps with the LPA peptide.
(TIF)

Figure S3 **FRET assays of GBS PI-1 SrtC1 and SrtC2 at different concentrations of peptide substrates.** Progress curves of the cleavage reaction of PI-1 (BP, AP1 and AP2) fluorescent peptides catalyzed by recombinant SrtC1 (top) and SrtC2 (bottom) wild type.
(TIF)
