# Supplementary figures and images for: Structural Basis for Group B Streptococcus Pilus 1 Sortases C Regulation and Specificity
Source: PLoS One. 2012 Nov 8;7(11):e49048. doi: 10.1371/journal.pone.0049048 (PMC3493515; doi:10.1371/journal.pone.0049048)

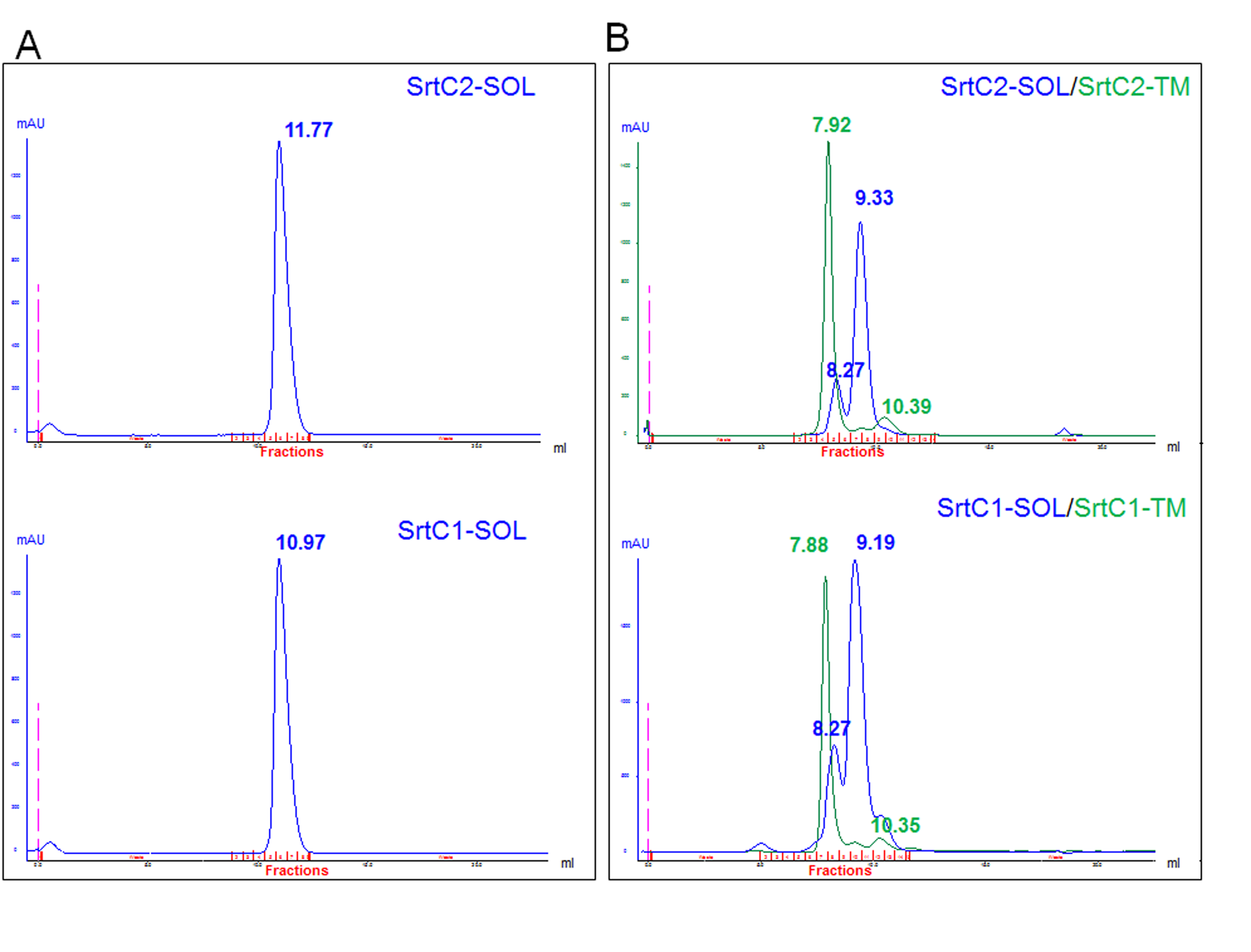

Supplement: Figure S1 — SEC analysis of recombinant SrtC2 and SrtC1. Recombinant enzymes run on a Superdex 75 10/300 SEC column connected to an Äkta Purifier. The Gel filtration standard (Biorad, 151-1901) was run on the same column and in the same buffer conditions: Thyroglobulin (670 kDa) elution volume 7.79 ml, γ-globulin (158 kDa) elution volume 8.10 ml, Ovalbumin (44 kDa) elution volume 9.92 ml, Myoglobin (17 kDa) elution volume 12.15 ml, Vitamin B12 (1.35 kDa) elution volume 18.17 ml. (A) The peaks at 11.77 ml and 10.97 ml correspond to monomeric SrtC2-SOL (predicted MW of 24 kDa) and SrtC1-SOL (predicted MW of 24.8 kDa) used for crystallization trials. (B) Superimposition of the chromatograms of soluble and TM containing sortases SrtC2-SOL/SrtC2-TM both fused with HIS-MBP (predicted MW 68.7 kDa and 71.4 kDa) and SrtC1-SOL/SrtC1-TM with HIS-MBP (predicted MW 69.5 kDa and 75.2 kDa), used for the FRET assays. The SrtC-TM proteins were prepared as HIS-MBP fusions in order to improve their solubility, as described in Materials and Methods. Here, the SrtC-SOL proteins were also prepared in HIS-MBP format, in order to allow a direct comparison with the SrtC-TM HIS-MBP proteins. Soluble sortases are mostly monomeric (blue chromatograms), eluting at the volumes of 9.33 ml and 9.19 ml. SrtC2-TM and SrtC2-TM (green chromatograms) eluting at the volumes of 7.92 ml and 7.88 ml are mostly aggregated, based on the standard. (TIF) [file pone.0049048.s001.tif]

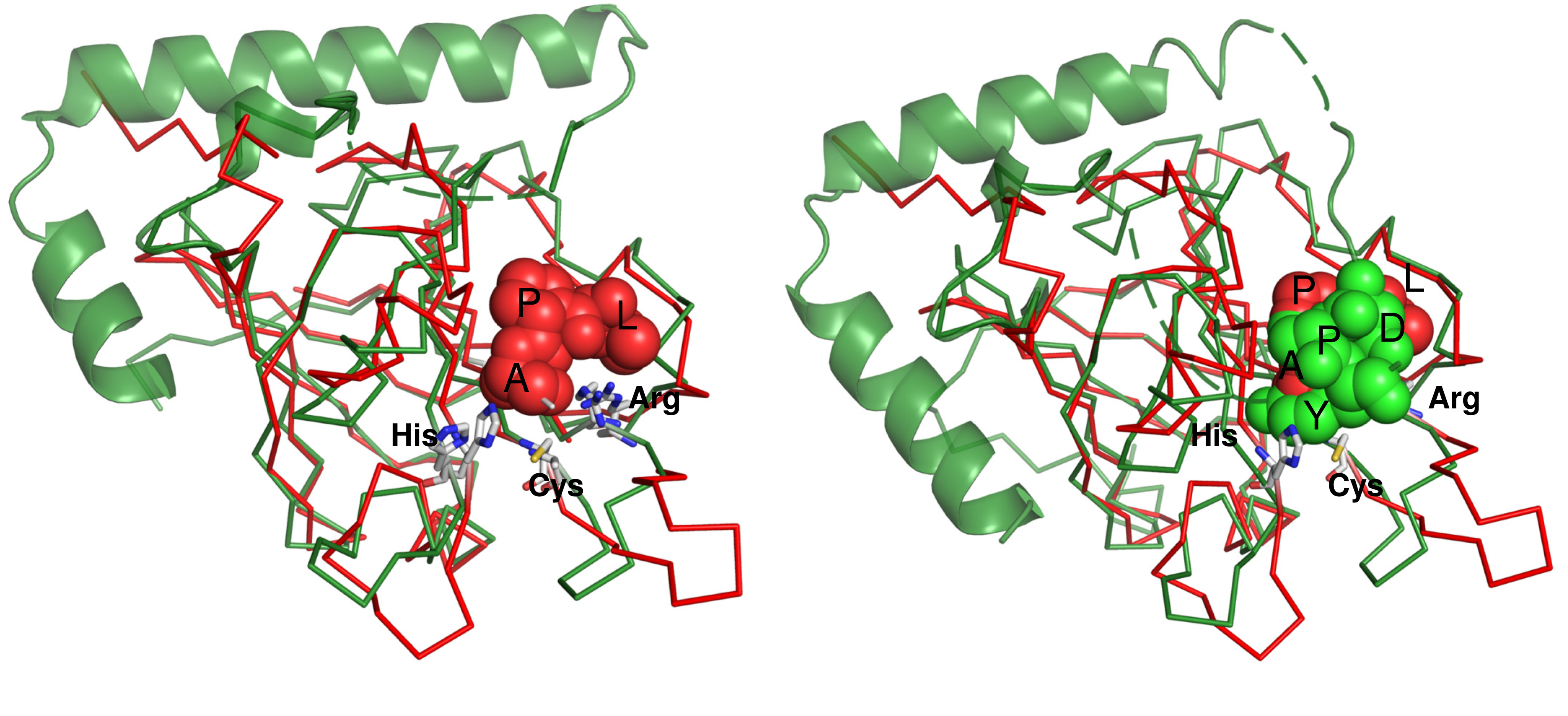

Supplement: Figure S2 — Superposition of the open-conformation (PDB 3RBJ) and close-conformation structures of GBS SrtC1-1 (in green) with S. aureus SrtA peptide-bound (red, PDB 2KID). In the open-conformation structure of GBS PI-1 SrtC1, the lid is displaced from the active site and the cleft is free to accommodate the LPA peptide (red spheres). On the contrary, in the close-conformation structure the conserved motif DPY (green spheres) in the SrtC1 lid overlaps with the LPA peptide. (TIF) [file pone.0049048.s002.tif]

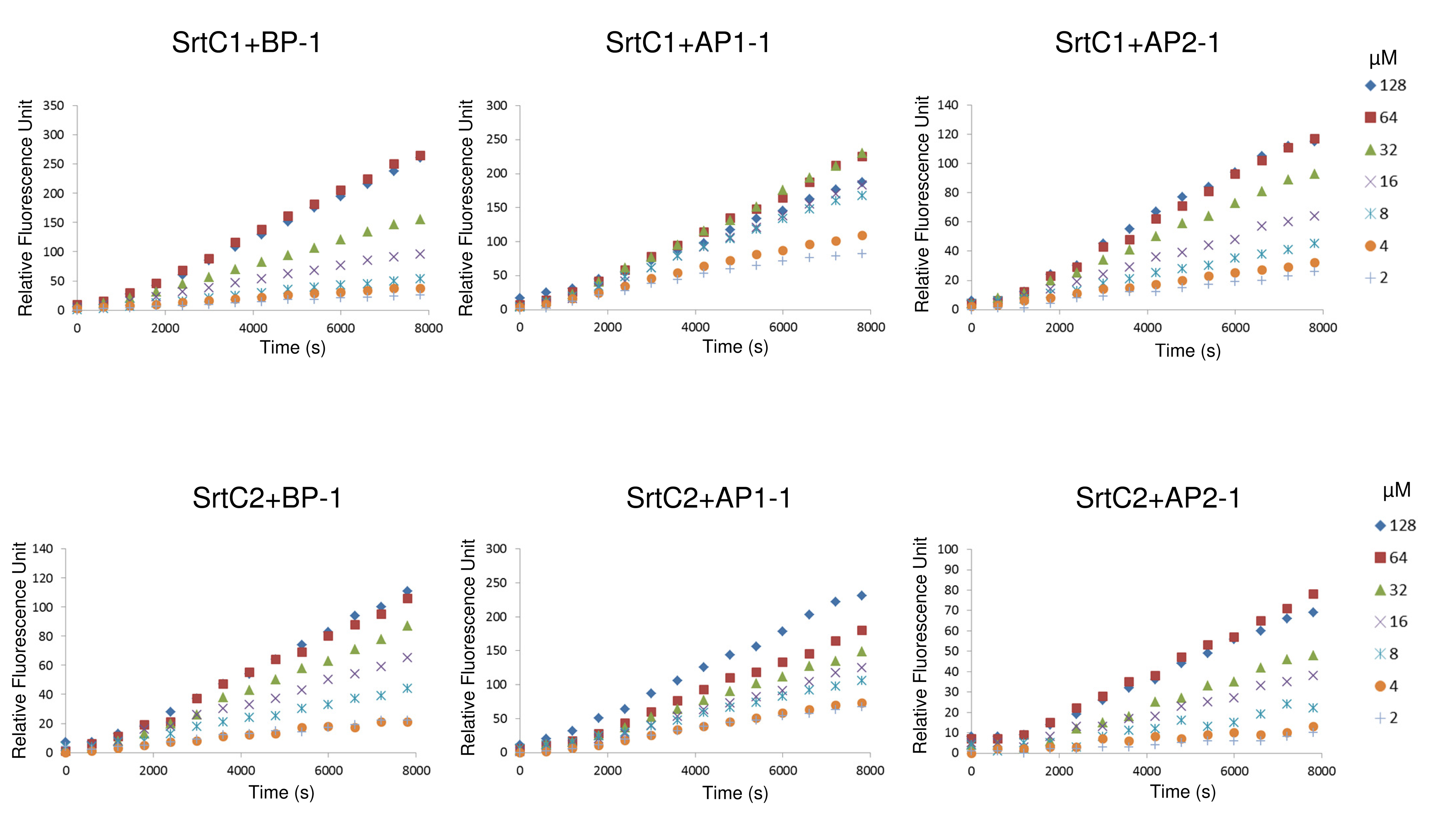

Supplement: Figure S3 — FRET assays of GBS PI-1 SrtC1 and SrtC2 at different concentrations of peptide substrates. Progress curves of the cleavage reaction of PI-1 (BP, AP1 and AP2) fluorescent peptides catalyzed by recombinant SrtC1 (top) and SrtC2 (bottom) wild type. (TIF) [file pone.0049048.s003.tif]
